# Comparison of a Web-Based 24-h Dietary Recall Tool (Foodbook24) to an Interviewer-Led 24-h Dietary Recall

**DOI:** 10.3390/nu9050425

**Published:** 2017-04-25

**Authors:** Claire M. Timon, Katie Evans, Laura Kehoe, Richard J. Blain, Albert Flynn, Eileen R. Gibney, Janette Walton

**Affiliations:** 1 Institute of Food and Health, University College Dublin, Belfield, Dublin 4, Ireland; claire.timon@ucd.ie (C.M.T.); rblain201@gmail.com (R.J.B.); 2 School of Food and Nutritional Sciences, University College Cork, Cork T12 YN60, Ireland; katie_evans1@yahoo.com (K.E.); laura.kehoe@ucc.ie (L.K.); A.Flynn@ucc.ie (A.F.); Janette.Walton@ucc.ie (J.W.)

**Keywords:** Foodbook24, dietary assessment, web-based, comparison, interviewer led, 24 h dietary recall, self-administered

## Abstract

Web-based tools have the potential to reduce the cost of dietary assessment; however, it is necessary to establish their performance compared to traditional dietary assessment methods. This study aims to compare nutrient and food intakes derived from Foodbook24 to those obtained from an interview-led 24-h dietary recall (24HDR). Seventy-nine adult participants completed one self-administered 24HDR using Foodbook24 and one interviewer-led 24HDR on the same day. Following a 10 days wash-out period the same process was completed again in opposite order to the previous study visit. Statistical analysis including Spearman’s rank order correlation, Mann-Whitney U tests, cross-classification analysis, and “Match”, “Omission”, and “Intrusion” rates were used to investigate the relationship between both methods. Strong, positive correlations of nutrient intake estimated using both methods was observed (*r*_s_ = 0.6–1.0; *p* < 0.001). The percentage of participants classified into the same tertile of nutrient intake distribution using both methods ranged from 58% (energy) to 82% (vitamin D). The overall match rate for food intake between both methods was 85%, while rates for omissions and intrusions were 11.5% and 3.5%, respectively. These results, alongside the reduced cost and participant burden associated with Foodbook24, highlight the tool’s potential as a viable alternative to the interviewer-led 24HDR.

## 1. Introduction

In recent years there has been a growing interest in the use of technology and web-based platforms in the assessment of dietary intake for large-scale dietary surveys. Technological advancements have led to the development of web-based, self-administered, 24-h dietary recalls (24HDR) which can aid in reducing the logistical burden and cost of conventional methods and may provide the opportunity for more efficient and cost-effective dietary assessments in comparison to more traditional paper-based methods [1,2,3,4,5]. Web-based tools can remove the need for interaction (face-to-face or over the phone) with a researcher, nutritionist, or dietitian, and may serve to reduce the influence of social desirability and, thus, encourage greater honesty when self-reporting intake [6]. Several web-based 24HDR tools have been successfully developed for different population groups in many countries, including the United Kingdom (UK) [1,2], France [3], Belgium [7], and the United States of America [4,5]. 

Although web-based tools may alleviate participant burden and attenuate some of the financial burdens associated with large-scale studies, it is vital that they are as accurate as the dietary assessment methods currently used in nutrition research and practice. A validation study is considered the most rigorous assessment of the accuracy of a test measure of dietary assessment and involves the performance of a test measure compared to an objective measure of intake, such as biological markers or direct observation of intake [8]. These types of investigations are not always financially feasible and rely largely on the willingness of participants to be observed or to provide biological samples. Additionally, validation studies do not always investigate how a test measure of dietary assessment compares to existing, commonly-used dietary assessment methodologies. Investigating the relative validity of a dietary assessment measure considers the performance of a ‘test measure’ against an alternate dietary recall (such as interviewer-led 24-h recall) or direct observation to assess the quality of nutrient and food group data collected by the test method compared to the existing method [9]. This is an important consideration as this type of study design allows the investigator to assess if the intuitive software design at the core of these web or computer based 24 HDR tools can actually achieve the same level of dietary intake assessment when compared to a trained interviewer. In the United States of America, The Automated Self-Administered 24-h Recall (ASA24), assessed in an adult population, has been shown to perform similarly to an interviewer-led 24HDR [10,11]. Bradley and colleagues [12] found that INTAKE24 (an online 24HDR tool) performed well compared to the interviewer-led 24HDR among 11–24 years old and suggested that web- or computer-based dietary assessment methods may be more engaging methods to use with children and adolescents compared to interviewer- or paper-based methodologies [12]. 

Foodbook24 is Ireland’s first web-based self-administered 24HDR tool and has been designed to collect food and nutrient intake data for the Irish adult population. Foodbook24 was developed by a team of nutritionists in University College Dublin (UCD) and University College Cork (UCC) in conjunction with software development experts from Creme Global. The aim of the present study was to compare a self-administered 24 HDR with a traditional interviewer-led dietary recall, by examining nutrient and food group intakes derived from both methods using the Foodbook24 tool as an entry method.

## 2. Materials and Methods

Ethical approval was obtained from the University College Cork Clinical Research Ethics Committee of the Cork Teaching Hospitals (ECM 4 (mm) 7 July 2015) and the University College Dublin Human Research Ethics Committee (LS 1526 Gibney-Timon).

### 2.1. Recruitment

A desired sample size of 80 participants was based on the number of participants recruited in similar investigations [11,13,14,15]. A convenience sample of 79 university students and staff were recruited via email, posters, and social media. Potential participants were screened for eligibility on first contact with the research team over the phone. Individuals who expressed an interest in the study were contacted by phone and screened for eligibility. Subjects were eligible if they were 18–64 years, fluent in English, had regular access to the Internet, were not pregnant or lactating, did not have any disease or condition that required chronic therapeutic nutritional or medical treatment, and had not been enrolled in or completed a degree or PhD in Human Nutrition. All participants were fully informed of the study requirements and were required to provide written consent before participating in the study.

### 2.2. Data Collection

Data collection took place between September and November 2015. Each participant was asked to complete both a self-administered 24HDR using Foodbook24 and an interviewer-led 24HDR on the same day on two separate occasions over a one month period. A total of 79 participants (40 female, 39 male) completed three study visits to either UCD or UCC. On the first visit participants provided informed written consent, completed a demographics questionnaire, and had height and weight measurements taken by the researcher. During study visit 2, participants completed an interviewer led 24HDR and a self-administered 24HDR using Foodbook24 on the same day. To investigate the impact of order of administration, a weighted randomisation of participants was employed with 75% of participants randomised to complete the web-based, self-administered 24HDR in the morning then subsequently completing an interviewer-led recall later that same day (a minimum three hours time-lapse). The remaining 25% completed the two recalls in the opposite order. Following a two week wash out period, participants completed two more recalls using the same methods, but in the opposite order to their previous visit. A total of twenty participants (ten in each study centre) completed their dietary recalls on a Monday to ensure weekend data was captured. Once participants completed the two recalls on two separate occasions they were asked to fill out a short online evaluation questionnaire in relation to the Foodbook24 tool.

### 2.3. Foodbook24

The Foodbook24 tool follows the design of the previously-published multi-pass recall model [16]. The initial pass requires the user to list all meals and snacks consumed the previous day, alongside the time of consumption and location of meal preparation. The user then adds individual food and drink components or composite dishes to each of the defined meals or snacks. The user can select these from a concise database of 751 food and beverage items and a number of built-in linked foods and prompt questions aid the participant in recalling items consumed in the previous 24 h period. Portion size information is then determined by selecting relevant amounts or portion size photographs and, lastly, the user is presented with a review of selected items, a list of frequently forgotten foods (such as beverages (including alcoholic beverages and water), sugar and sweetener (added to beverages or cereal), spreads, condiments, biscuits, confectionaries, fruit, and bread) and queried about nutritional supplement intake. The concise food list integrated into the Foodbook24 tool was developed using nationally-representative food consumption data and food grouping structure from the National Adult Nutrition Survey (NANS) (2008–2010) [17]. Both the development of the tool itself and the establishment of the shortened food list for integration into the tool have been described elsewhere [18,19]. The NANS food composition database used data from McCance and Widdowson’s The Composition of Foods, sixth and fifth editions, plus all nine supplemental volumes to generate nutrient intake data. Modifications were made to the food composition database to include recipes of composite dishes, fortified foods, and foods specific to the Irish market.

### 2.4. Data Entry

For the self-administered 24HDR participants entered all food and drinks consumed the previous day (from midnight to midnight) into the Foodbook24 tool using free text entry to search for the respective food and drinks in the database. Once the food is selected, the participant estimates the portion size with the aid of portion size images that are incorporated into the Foodbook24 tool. The tool automatically codes the recall and assigns the nutritional composition. For the interviewer-led dietary recall, a 24HDR was conducted in person using the US Department of Agriculture Automated Multiple-Pass Method. Trained researchers conducted the 24-h dietary recall using the five-step protocol described by Moshfegh and colleagues [16] and a printed version of the Foodbook24 portion sizes was used to aid portion size quantification. The photographs used in the software and in the printed version had the same sized dimensions and included a knife and a fork on either side of the plate as a standard of reference. The information recalled was documented traditionally (on paper) and then researchers followed a structured protocol to enter the collected data in the interview back into the Foodbook24 tool. The structured coding protocol was adhered to by all researchers in an attempt to maintain consistency and all entered data was reviewed independently by a separate researcher. Therefore, the data was analysed in two separate ways, firstly, the self-administered 24HDR were automatically analysed by the tool, itself, and, secondly, the interviewer-led recall was entered into Foodbook24 tool by a researcher. 

### 2.5. Statistical Analysis

All statistical analysis was computed in IBM SPSS Statistics (version 21) (Armonk, NY, USA). Normality of data was investigated using the Kolmogorov-Smirnov test. All data was not normally distributed, meaning non-parametric tests were applied for statistical analysis. Mean daily intake of energy and a wide range of nutrients, including protein, carbohydrate, total fat and fatty acids, and dietary fibre and micronutrients, including vitamins A, D, E, C, and B vitamins and calcium, magnesium, iron, phosphorus, copper, zinc, potassium, and sodium were estimated for both the self-administered and interviewer-led 24HDR. The difference in self-reported energy and nutrient intakes were examined by calculating the difference in each individual’s intakes derived from each recall, for each day recorded (Recall 1 and 2 were analysed separately). Mann-Whitney U tests were performed to identify significant differences between estimates. Differences were considered significant at *p* < 0.01 to compensate for a possible increased risk of Type 1 errors arising from multiple tests. Spearman’s rank order correlations were performed to assess the relationship between estimates of nutrient intake between the self-administered and the interviewer-led recall for the same day. Cross-classification analysis was performed to quantify the level of agreement between the categorisation of estimates (from the self-administered recall and the corresponding interviewer-led recall) into thirds of the intake distribution. The percentage of participants categorised into the same tertile of intake was calculated.

Bland and Altman analysis was performed to assess the limits of agreement in the reporting of nutrient intake, considering the two methods of dietary assessment to be comparable if greater than 95% of the data plots were within the limits of agreement. 

Analysis by food groups was also conducted. Twenty eight food groups were selected e.g., breads and rolls, breakfast cereals, and beverages, and daily intake for each food group was examined for Recall 1 and 2 using both the self-administered recall and the corresponding interviewer-led recall. The percentage difference between the corresponding recalls was calculated and, similarly to the nutrient intake analysis, Mann-Whitney U tests were performed to identify if the differences between estimates were significant (*p* < 0.01). Spearman rank order correlations and cross-classification analyses were performed to quantify the level of agreement between the categorisation of estimates (from corresponding recalls) into thirds of intake distribution and to calculate the percentage of participants categorised into the same tertile of food group intake. Bland and Altman analysis was performed to assess the limits of agreement in the reporting of food group intake, considering the two methods of dietary assessment to be comparable if greater than 95% of the data plots were within the limits of agreement. 

For the purpose of this analysis and the limited sample size (*n* = 79) under-reporters were not excluded.

### 2.6. Matches, Omissions, and Intrusions

Match, omission, and intrusion rates were calculated to assess the agreement of the self-administered dietary recall with the interviewer-led recall. A “match” was defined as a selection of the exact same food or drink item in both the self-administered and interviewer-led 24HDR, while an “approximate match” was defined as the same food but a slightly different variation of that food, i.e., skimmed milk and whole milk, or porridge made with water and porridge made with whole milk. An “omission” was defined as a food or drink item reported in the interviewer-led recall but not in the self-administered recall whilst an “intrusion” was defined as a food or drink item reported in the self-administered recall but not in the interviewer-led recall. Chi-squared analysis was additionally used to assess differences in the incidences of matches, omissions, and intrusions between males and females.

## 3. Results

A total of 95 participants signed up for the present study (41 in UCC and 54 in UCD). Eight participants were ineligible to take part due to unsatisfactory English (*n* = 3) and prior nutritional education (*n* = 5), while a further four participants dropped out of the study citing time commitments as an issue. Additionally, four participants were excluded from the final analysis as they did not follow the study protocol correctly. The final analysis was carried out on 79 participants (40 Female, 39 Male). Table 1 displays the demographic characteristics of the final sample (*n* = 79). The mean age of the sample was 33 years old (age range of 18–60 years). The majority of participants were Caucasian (93.7%) and were either staff (41.8%) or students (32.9%) at UCD or UCC, with the remaining participants employed outside of the university (25.3%). The majority were non-smokers (78.5%) and most reported having no medical conditions (81%).

Table 2 presents the mean daily intake of energy, dietary fibre, and macro- and micro-nutrients, as recorded by the self-administered recall and interviewer-led recall for Recall 1 and 2. Overall, the self-administered dietary recall estimated a lower mean daily intake for energy and the majority of nutrients compared to the interviewer-led recall, with the exception of carotene for Recall 1 and carotene and vitamin C for Recall 2, although these were not statistically significant. 

The mean difference between estimates of intake derived from each method were calculated and presented as mean percentage difference. For Recall 1, the percentage difference between both methods was within −10% to +10% for 11 nutrients (carbohydrate, starch, dietary fibre, carotene, vitamin D, total niacin, pantothenate, vitamin C, magnesium, iron, and sodium). The most notable mean percentage differences arose from lower intakes in the self-administered 24HDR for vitamin B1, retinol, saturated fat, total fat, and monounsaturated fat (ranging from 20% to 30% less). For Recall 2, the percentage difference between both methods within −10% to +10% increased to 25 nutrients for Recall 2 (protein, carbohydrate, total sugars, non-milk sugars, starch, dietary fibre, polyunsaturated fat, carotene, vitamins D, E, B1, total niacin, B6, B12, folate, folic acid, pantothenate, vitamin C, magnesium, phosphorus, iron, copper, zinc, potassium, and sodium). For Recall 2, the largest mean percentage differences arose from lower intakes in the self-administered 24HDR for calcium, saturated fat, vitamin B2, and monounsaturated fat (ranging from 17% to 21% less). However, none of the differences observed were statistically significant, except for energy and total fat, which were higher in the interviewer-led recall for both Recall 1 and 2.

The association between estimates of nutrient intake was assessed using Spearman’s rho (r_s_) for rank correlation and by calculating the percentage of participants who were categorised into the same and opposite thirds of the intake distribution based on the self-administered and corresponding interviewer-led recall (Table 3). For both Recall 1 and 2, a strong positive association between the estimates of daily nutrient intake from each method (*r*_s_ = 0.6–1.0, *n* = 79, *p* < 0.001) was observed. The rank correlations between the self-administered and interviewer-led recalls ranged from 0.617 for energy (kcal) to 0.976 for folic acid for Recall 1, and from 0.566 for saturated fat to 0.893 for folic acid for Recall 2. With the exception of folic acid (32%), the percentage of participants classified into the same third of the nutrient intake distribution when using the self-administered and corresponding interviewer-led recall for Recall 1 was >50%, ranging from 58% for energy (kcal) to 82% for vitamin D. For Recall 2, the percentage of participants classified into the same third of the nutrient intake distribution was >50%, ranging from 54% for copper to 95% for folic acid.

Similar analysis was conducted for food group intakes with the mean daily intake as recorded by the self-administered recall and interviewer-led recall for Recall 1 and 2 presented in Table 4. The average number of foods reported by the self-administered and the interviewer-led recalls were 18 (range; 5–31) and 22 (range; 10–41) for Recall 1 and 19 (range; 5–34) and 22 (range; 10–40) for Recall 2, respectively (data not shown). The self-administered dietary recall generally estimated a lower or similar mean daily intake for most food groups. For Recall 1, the percentage difference between both methods was within −10% to +10% for six of the food groups (“potato and potato dishes”, “fruit and fruit dishes”, “fish and fish dishes”, “meat and meat dishes”, “chocolate confectionary”, and “carbonated beverages”). The largest percent differences observed in Recall 1 were for non-chocolate confectionery, water, “squashes and cordials”, and teas (ranged from 51% to 136% decrease in daily intake using the self-administered tool). Smaller differences in food group intakes were observed for Recall 2 and the percentage difference between both methods within −10% to +10% increased to nine food groups (“grains, rice, pasta and savouries”, “breakfast cereals”, “creams, ice cream and chilled desserts, “egg and egg dishes”, “potato and potato dishes”, “fruit and fruit dishes”, “fish and fish dishes”, “meat and meat dishes”, and “nuts, seeds, herbs and spices”). The largest were observed for “cheeses”, “water”, “butter, spreading fats and oils”, and “savoury snacks” (ranged from 80% to 50% decrease using the self-administered method). However the differences observed were not significant with the exception of water in Recall 1 and 2 (*p* = 0.000) (Table 4).

The rank correlations of food group intakes between the self-administered and interviewer-led recalls ranged from *r*_s_ = 0.520–0.941 for “fish and fish dishes” and “coffees”, respectively, for Recall 1 and from *r*_s_ = 0.461–0.950 for “water” and “breakfast cereals”, respectively, for Recall 2 (Table 5). The percentage of participants who were categorised into the same third of food group intake distribution based on the self-administered and corresponding interviewer-led recall were similar for both recalls. For Recall 1 this ranged from 24% for cheeses to 100% for fish and fish dishes and for Recall 2 ranged from 25% for cheeses to 98% for squashes and cordials and alcoholic beverages (Table 5). However, as there were low numbers of consumers (<30%) for some food groups such as “creams, ice-creams and chilled desserts”, “eggs and egg dishes”, “fish and fish dishes” “non-chocolate confectionery”, “savoury snacks”, “alcoholic beverages”, “carbonated beverages” and “squashes” the results should be interpreted with caution. 

Table 6 displays the results of the matches, omissions and intrusions analysis. Recall 1 and 2 were examined separately and overall the percentage of foods reported as matches, omissions and intrusions were similar. The percentage of omissions observed was similar in both recalls (12% in Recall 1 and 11% in Recall 2) while intrusions were also similar in both recalls (4% in Recall 1 and 3% in Recall 2). Chi-squared analysis was additionally used to assess the differences in the incidences of matches, omissions, and intrusions between males and females, which highlighted that matches were highest amongst women, with men more likely to omit food items when recording intake using Foodbook24 (*p* = 0.000).

## 4. Discussion

This study aimed to investigate whether Foodbook24 performs as well, or comparably well, to an interviewer-led 24HDR. The design and development of Foodbook24 is described in detail elsewhere [19]. Overall, Foodbook24 estimated lower intakes of energy and some nutrients compared to the interviewer-led 24HDR. However, energy, total fat, and monounsaturated fats were significantly different between both methods. Recent studies looking at the comparability of web-based recall tools and interviewer-led recalls have reported similar findings. Foster et al. [1] noted a 3% underestimation of energy intake by INTAKE24, while Albar and colleagues [14] also found that ‘myfood24’ underestimated intakes of energy across two recalls among adolescents. Biltoft-Jensen et al. [20] compared energy intake derived from WebDASC (an interactive food record-recall tool) against TEE among children aged 8–11 years old, and reported good agreement between both methods at a group level, but noted substantial variation at an individual level. In contrast, Thompson et al. [10] reported energy and nutrient intake estimates to be equivalent between ASA24 and an interviewer-led 24HDR. The mean percentage difference of nutrients was lower in Recall 2 and may highlight an increased understanding on the part of the participants in Recall 2. Having completed Recall 1, it is possible that participants became more proficient in using the tool. The low levels of agreement for folic acid suggest that improvements to the tool may be required to assess intakes of episodically consumed nutrients. 

Mean intakes of food groups between both methods varied. As mentioned, in Recall 2 the number of food groups with percentage differences between ±10% estimated by both methods increased compared to Recall 1, again highlighting participants becoming increasingly proficient using the tool in Recall 2. In particular, there were small percentage differences for ‘fruit and fruit dishes’, ‘vegetables and vegetable dishes’, ‘fish and fish dishes’, and ‘potatoes and potato dishes’, while there were some larger percentage differences for water, squashes, and cordial type drinks, and non-chocolate confectionary. Through entering the self-administered data back into Foodbook24, the researchers became aware of a common trend of participants underestimating or omitting water altogether in the self-administered 24HDR. The under reporting of water intake using web based tools was also noted in other comparison studies [21]. This may have been the result of participants consuming water throughout the day in small quantities, thus making it harder to recall. Perhaps a dedicated prompt relating to water consumption could be implemented in future versions of the tool. Additionally, dedicated prompts may be required for snack foods such as nuts and chocolate confectionary to improve recall of these foods which would typically be prompted in a traditional interview-led recall. However, water was the only significantly different food group across both recalls, suggesting that overall food group intakes recorded using the self-administered approach may be similar to those obtained via the interviewer-administered approached

Analysis of the incidence of matches, omissions and intrusions of foods and drinks between the self-administered and interviewer-led 24HDR showed an exact match rate of 69%, with a further 18% deemed approximate matches, 11% omissions, and 3% intrusions across Recall 1 and 2. ‘Condiments’, ‘herbs and spices’, ‘cream’, ‘seeds’, and ‘water’ were the most frequently omitted food items using Foodbook24. Few studies have employed the same methodology as the present study with the majority only reporting three classifications; matches, omissions, and intrusions and not approximate matches. Foster et al. [1] reported both exact match (80%) and approximate match rates (1.3%) between INTAKE24 and an interviewer-led 24HDR among adolescents (11–16 years) and young adults (17–24 years), while omissions (10.7%) and intrusions (7.1%) were also similar to the present study. Interestingly, female participants had higher incidence of matches between both recalls, where male participants were more likely to omit food and drink items, which has also been reported in other studies [22]. 

Composite dishes were a contributing factor to the high approximate match rate in the present study. Upon analysing the data, the researchers became aware that users were more inclined to report composite dishes as one entry in the self-administered 24HDR, as opposed to multiple components in the interviewer-led 24HDR. For example, in the self-administered recall, a participant may search for and select ‘Spaghetti Bolognese’ from the database as one entry and use the associated portion size image to quantify the portion size. However, in the interviewer-led recall, with prompts and probes from the researcher, the participant may have been more expansive, listing multiple components of the dish. Similar to the findings in this present study, Kirkpatrick et al. [11] also identified additions to, or ingredients, in multicomponent/composite dishes as the most common exclusions in the investigation of the performance of ASA24. Although this may influence the exact match rate between both methods, at a population level, the reduction in participant burden associated with simply selecting one composite dish or meal, may offset the small effect it has on accuracy, as demonstrated by Woolhead et al. [23] whereby the potential of the generic meal approach to examine nutrient intakes at a population level was highlighted. However, this approach may not be suitable for the investigation of individual level dietary intake data for certain epidemiologic or clinical studies.

As mentioned, few studies investigating matches, omission, and intrusion rates have used the same methods as the present study. Much of the research surrounding web-based tools and food group match rates have been carried out with younger age groups compared to the adult sample in the present study. Previous research investigating web-based tools among children has yielded mixed results. Using a mix of direct observation and interviewer-led 24HDR as methods, several studies have reported similar rates for matches (48–76%), omission (15–24%), and intrusions (9–15%) [13,24,25,26]. Many of the researchers in question cited the age of participants as a determining factor of recall accuracy, which would explain the contrast to the present study. Some studies noted there was assistance made available to children enrolled in their respective study [24,27], while others did not state whether there was researcher assistance involved. Baranowski et al. [25] set out to determine the age at which accuracy is most influential when using such web-based tools and, as such, no assistance was given to the children. The low match rate (48%) reflects this lack of support. In the present study, participants (18+ years) received no training on how to use Foodbook24. Although a satisfactory match rate was achieved, overall, there is a possibility that, with prior training, users could be more proficient in using the tool.

## 5. Strengths and Limitations

In terms of study strengths, the study design ensured that both methods were collecting intake data for the same period of time. In addition, each participant completed two 24HDR with both methods, while a percentage of the sample completed their recall on Monday, ensuring weekend intake was captured. Lastly, a weighted randomisation was employed so the researchers could observe any learning effect which may have influenced the accuracy of Foodbook24 at collecting intake data. A limitation of the study is that the convenience sample was comprised of university students and staff. This sample may not represent the wider community and may also have a higher than average computer literacy which is a defining factor in the potential of web-based tools [28]. As the sample size was limited, low energy reporters were not excluded from the analysis, which is likely to explain the low mean daily energy intakes recorded during the study. It is also important to note that in this present study the same food and nutrient composition database was used to analyse both sets of data (self-administered and interviewer administered recall data), which is not always the case in other studies. 

## 6. Conclusions

Foodbook24 is a web-based 24HDR tool specifically developed for the Irish adult population and consists of nationally-representative food intake data. This study has shown that a self-administered 24HDR using Foodbook24 compares well to the currently-used interviewer-led 24HDR utilising the Foodbook24 tool as an entry method. Further development to increase the current food and drink list may reduce some of the discrepancies observed between the methods in the reporting of certain nutrient intakes. That being said, the potential of remote access and freedom to complete recalls at the user’s leisure renders Foodbook24 an attractive alternative to face-to-face consultations. Such advantages may encourage participation rates in longitudinal epidemiological studies which may yield valuable intake data at a population level. The results of this study highlight the potential of Foodbook24 as a viable alternative to traditional dietary assessment methods for researchers in Ireland.

## Figures and Tables

**Table 1:** Demographic characteristics of participants.

**Gender, Age and BMI**	
Female (*n*)	40
Male (*n*)	39
Age (Mean, SD) (years)	33.15 (12.46)
Age range	18–60 years
BMI ^a^ (kg/m^2^) (Mean, SD)	24.52 (3.64)
**Ethnicity**	(%)
Caucasian	93.7
Other	6.4
**Occupation**	(%)
University staff	41.8
Student	32.9
Employed outside of the University	25.3
**Smoking habits**	(%)
Non-smoker	78.5
Smoker	11.4
Ex-smoker	10.1
**Medical conditions:**	(%)
None	81
One or more	19

^a^ Body mass index (BMI); SD: Standard deviation.

**Table 2:** Daily energy and nutrient intakes recorded by participants using the Foodbook24 tool and interviewer led 24-h recall.

Nutrient	Recall 1								Recall 2							
	Self-Administered			Interviewer-Led					Self-Administered			Interviewer-Led				
	Mean	Median	SD	Mean	Median	SD	Percentage Difference ^ⱡ^	*p*	Mean	Median	SD	Mean	Median	SD	Percentage Difference ^ⱡ^	*p*
Energy (kcal)	1888	1757	743	2168	1986	853	−15	0.008	1817	1737	670	2019	1922	639	−11	0.023
Protein (g)	77	68	37	88	79	37	−14	0.030	79	72	34	86	81	31	−8	0.148
Carbohydrate (g)	226	208	95	247	228	107	−9	0.156	216	197	104	233	212	96	−8	0.129
Total sugars (g)	94	83	51	109	89	63	−17	0.084	99	82	76	105	93	64	−6	0.122
Non-milk sugars (g)	81	73	48	93	79	59	−15	0.171	89	75	74	91	82	63	−2	0.326
Starch (g)	126	114	68	131	121	72	−4	0.589	111	106	52	122	118	54	−10	0.228
Dietary fibre (g)	22	20	10	24	22	9	−7	0.272	22	21	11	23	23	8	−4	0.283
Total fat (g)	73	67	35	88	80	39	−20	0.007	70	62	32	81	75	35	−16	0.040
Saturated fat (g)	29	26	17	35	33	19	−24	0.013	28	25	14	33	30	18	−21	0.050
Monounsaturated fat (g)	27	25	14	33	30	16	−20	0.013	26	23	13	30	29	13	−17	0.025
Polyunsaturated fat (g)	13	11	7	15	13	8	−19	0.071	13	12	7	13	13	6	−4	0.439
Retinol (µg)	301	223	284	390	322	288	−30	0.015	320	245	262	366	323	251	−14	0.147
Carotene (µg)	4429	2367	6648	4285	2974	4951	3	0.383	5173	2551	7199	4828	2668	6180	7	0.825
Vitamin D (µg)	2.9	1.9	2.8	3.1	2.1	2.9	−9	0.587	2.4	1.8	2.2	2.4	2.0	2.0	−2	0.692
Vitamin E (mg)	10.4	10.1	5.0	11.5	11.2	5.1	−11	0.134	10.0	9.2	5.2	10.5	9.4	5.4	−5	0.512
Vitamin B1 (mg)	1.6	1.4	1.1	2.1	1.4	2.8	−30	0.199	1.4	1.4	0.6	1.5	1.3	0.7	−7	0.368
Vitamin B2 (mg)	1.6	1.4	0.9	1.9	1.7	1.0	−18	0.033	1.4	1.3	0.7	1.7	1.6	0.8	−19	0.023
Total niacin (mg)	38.2	32.2	20.4	41.6	39.6	19.7	−9	0.173	39.8	34.1	23.8	39.7	37.2	16.3	0	0.572
Vitamin B6 (mg)	2.1	1.9	1.3	2.4	2.2	1.3	−11	0.081	2.5	1.9	3.9	2.2	2.0	1.0	10	0.273
Vitamin B12 (µg)	4.3	3.2	3.5	5.1	3.6	4.3	−18	0.254	4.1	2.8	5.0	4.2	3.5	3.1	−2	0.273
Folate (µg)	258	235	117	292	255	135	−13	0.124	261	232	132	278	268	128	−6	0.312
Folic acid (µg)	35	0	66	39	0	74	−11	0.867	30	0	64	33	0	67	−9	0.901
Biotin (µg)	37.4	34.1	17.4	43.8	41.1	21.3	−17	0.036	35.7	30.6	21.4	41.9	37.7	20.8	−17	0.026
Pantothenate (mg)	5.9	5.3	3.5	6.6	6.2	3.6	−10	0.124	5.6	4.9	4.4	5.7	5.4	2.4	−1	0.186
Vitamin C (mg)	116	87	109	120	85	116	−3	0.674	117	88	111	114	85	105	2	0.829
Calcium (mg)	863	740	457	1024	900	539	−19	0.046	802	755	377	971	951	452	−21	0.015
Magnesium (mg)	294	287	103	324	312	106	−10	0.058	296	287	133	314	301	98	−6	0.084
Phosphorous (mg)	1337	1247	536	1495	1353	545	−12	0.060	1315	1261	501	1441	1441	454	−10	0.046
Iron (mg)	11.6	11.3	5.5	12.7	11.7	6.0	−9	0.185	11.2	10.2	4.7	12.1	11.0	4.4	−8	0.13
Copper (mg)	1.1	1.1	0.4	1.2	1.2	0.5	−11	0.117	1.1	1.0	0.5	1.2	1.1	0.4	−6	0.172
Zinc (mg)	8.5	8.3	3.8	10.0	9.0	4.3	−17	0.023	8.7	7.8	4.6	9.3	8.4	3.5	−7	0.093
Potassium (mg)	3045	2915	1162	3388	3243	1265	−11	0.067	2902	2780	1037	3102	2940	940	−7	0.179
Sodium (mg)	2566	2254	1624	2583	2364	1476	−1	0.655	2168	2108	994	2358	2200	1103	−9	0.371

^ⱡ^ Calculated as the difference of the mean intake (self-administered- interviewer-led) divided by the mean intake (self-administered) and multiplied by 100.

**Table 3:** Association between the estimates of nutrient intake from self-administered and interviewer-led recalls split by recall number.

Nutrient	Recall 1						Recall 2					
	Spearman Correlations		% in the Same Tertile	Difference in Mean Daily Intake (*p*)	Mean Difference	Limits of Agreement ^†^	Spearman Correlations		% in the Same Tertile	Difference in Mean Daily Intake (*p*)	Mean Difference	Limits of Agreement ^†^
	*r*	*p*	%				*r*	*p*	%			
Energy (kcal)	0.617	<0.001	58.2	<0.001 **	−280.16	−389.89/−170.43	0.717	<0.001	64.6	0.001 **	−202.42	−315.47/−89.36
Protein (g)	0.772	<0.001	73.4	<0.001 **	−10.75	−15.26/−6.24	0.789	<0.001	74.7	0.013	−6.30	−11.26/−1.34
Carbohydrate (g)	0.651	<0.001	58.2	0.006 *	−20.46	−34.82/−6.10	0.798	<0.001	70.9	0.019	−16.95	−31.00/−2.90
Total sugars (g)	0.691	<0.001	67.1	0.002 *	−15.76	−25.38/−6.13	0.781	<0.001	67.1	0.271	−5.68	−15.88/4.52
Starch (g)	0.812	<0.001	69.6	0.203	−5.10	−13.01/2.80	0.874	<0.001	70.9	<0.001 **	−10.86	−16.73/−5.00
Dietary fibre (g)	0.796	<0.001	68.4	0.043	−1.49	−2.92/−0.05	0.727	<0.001	68.4	0.403	−0.82	−2.76/1.12
Total fat (g)	0.749	<0.001	60.8	<0.001 **	−14.92	−19.83/−10.01	0.610	<0.001	64.6	0.001 **	−11.14	−17.82/−4.46
Saturated fat (g)	0.763	<0.001	67.1	<0.001 **	−6.85	−9.31/−4.39	0.566	<0.001	55.7	0.001 **	−5.82	−9.08/−2.56
Monounsaturated fat (g)	0.777	<0.001	62.0	<0.001 **	−5.44	−7.29/−3.60	0.602	<0.001	62.0	0.001 **	−4.41	−7.04/−1.78
Polyunsaturated fat (g)	0.698	<0.001	69.6	<0.001 **	−2.46	−3.68/−1.25	0.671	<0.001	63.3	0.333	−0.56	−1.71/0.59
Retinol (µg)	0.718	<0.001	59.5	<0.001 **	−89.11	−128.18/−50.04	0.674	<0.001	60.8	0.028	−45.84	−86.70/−4.97
Carotene (µg)	0.860	<0.001	81.0	0.671	144.19	−528.44/816.82	0.843	<0.001	78.5	0.427	345.73	−515.52/1206.98
Vitamin D (µg)	0.908	<0.001	82.3	0.115	−0.25	−0.57/0.06	0.721	<0.001	64.6	0.799	−0.04	−0.39/0.30
Vitamin E (mg)	0.773	<0.001	64.6	0.006*	−1.12	−1.91/−0.34	0.755	<0.001	67.1	0.238	−0.47	−1.27/0.32
Vitamin B1 (mg)	0.703	<0.001	63.3	0.060	−0.48	−0.98/0.02	0.743	<0.001	67.1	0.028	−0.10	−0.19/−0.01
Vitamin B2 (mg)	0.764	<0.001	73.4	<0.001 **	−0.30	−0.42/−0.17	0.687	<0.001	62.0	<0.001 **	−0.27	−0.40/−0.14
Total niacin (mg)	0.805	<0.001	73.4	0.006 *	−3.43	−5.85/−1.01	0.760	<0.001	72.2	0.962	0.11	−4.35/4.57
Vitamin B6 (mg)	0.743	<0.001	68.4	0.002 *	−0.24	−0.38/−0.09	0.689	<0.001	68.4	0.557	0.26	−0.61/1.12
Vitamin B12 (µg)	0.767	<0.001	63.3	0.001 **	−0.79	−1.25/−0.33	0.766	<0.001	74.7	0.891	−0.07	−1.05/0.91
Folate (µg)	0.762	<0.001	67.1	0.001 **	−34.33	−54.93/−13.73	0.731	<0.001	62.0	0.124	−16.85	−38.41/4.71
Folic acid (µg)	0.976	<0.001	31.6	0.159	−3.94	−9.46/1.57	0.893	<0.001	94.9	0.265	−2.82	−7.82/2.18
Vitamin C (mg)	0.684	<0.001	63.3	0.793	−3.16	−27.11/20.78	0.860	<0.001	72.2	0.719	2.67	−12.08/17.43
Calcium (mg)	0.776	<0.001	62.0	<0.001 **	−160.93	−228.82/−93.05	0.689	<0.001	65.8	<0.001 **	−168.45	−240.63/−96.27
Magnesium (mg)	0.737	<0.001	64.6	0.001 **	−30.03	−46.49/−13.56	0.678	<0.001	65.8	0.172	−18.19	−44.47/8.09
Phosphorous (mg)	0.727	<0.001	68.4	<0.001 **	−158.28	−230.44/−86.12	0.727	<0.001	64.6	0.005	−126.22	−212.52/−39.92
Iron (mg)	0.781	<0.001	72.2	0.003 *	−1.11	−1.83/−0.39	0.674	<0.001	60.8	0.043	−0.90	−1.78/−0.03
Copper (mg)	0.729	<0.001	65.8	0.001 **	−0.12	−0.18/−0.05	0.605	<0.001	54.4	0.190	−0.07	−0.18/0.04
Zinc (mg)	0.766	<0.001	70.9	<0.001 **	−1.48	−2.04/−0.93	0.773	<0.001	72.2	0.135	−0.65	−1.50/0.20
Potassium (mg)	0.719	<0.001	68.4	0.001 **	−342.53	−543.85/−141.22	0.699	<0.001	64.6	0.021	−200.44	−370.29/−30.59
Sodium (mg)	0.746	<0.001	70.9	0.898	−16.76	−276.30/242.79	0.628	<0.001	64.6	0.041	−189.94	−372.11/−7.77

* = significant at the 0.01 level; ** = significant at the <0.001 level; ^†^ Lower and upper limits of agreement (mean difference ± 2 SD (standard deviation)).

**Table 4:** Mean intake of food groups (g/day) for self-administered and interviewer-led recalls split by recall number.

Food Groups	Recall 1								Recall 2							
	Self-Administered			Interviewer-Led					Self-Administered			Interviewer-Led				
	Mean	Median	SD	Mean	Median	SD	Percentage Difference ^ⱡ^	*p*	Mean	Median	SD	Mean	Median	SD	Percentage Difference ^ⱡ^	*p*
Grains, rice, pastas and savouries	126	106	160	93	0	123	26	0.236	91	0	127	82	0	117	9	0.940
Bread and rolls	74	55	89	90	64	101	−21	0.250	76	64	70	98	82	77	−28	0.069
Breakfast cereals	88	38	104	98	52	113	−11	0.561	75	38	99	77	38	93	−3	0.696
Biscuits, cakes and pastries	33	16	39	41	20	52	−23	0.609	34	15	44	38	18	55	−12	0.878
Milk and yoghurt	200	100	268	247	100	330	−23	0.557	169	100	201	205	125	249	−21	0.466
Milks	156	100	232	197	100	291	−26	0.574	133	0	202	166	0	244	−24	0.545
Creams, ice creams and chilled desserts	13	0	36	17	0	47	−32	0.355	9	0	30	9	0	30	5	0.820
Cheeses	10	0	18	14	0	24	−45	0.200	11	0	19	20	0	29	−80	0.054
Butter, spreading fats and oils	8	0	15	10	5	14	−28	0.114	8	5	12	13	8	21	−66	0.078
Other dairy	67	20	114	81	30	113	−21	0.156	55	20	85	67	40	86	−20	0.165
Eggs and egg dishes	29	0	51	36	0	62	−25	0.600	31	0	56	30	0	58	3	0.835
Potatoes and potato products	63	0	98	64	0	94	−2	0.979	54	0	85	57	0	92	−6	0.892
Veg and veg dishes	142	134	132	150	128	119	−6	0.477	151	101	164	168	115	164	−11	0.275
Fruit and fruit juices	259	194	244	273	196	300	−5	0.928	269	208	287	249	196	256	7	0.835
Fish and fish dishes	26	0	63	25	0	54	5	0.965	20	0	56	22	0	56	−8	0.675
Meat and meat products	155	125	168	175	152	188	−13	0.371	172	159	168	163	146	150	5	0.930
Sugars, confectionery, preserves and savoury snacks	27	15	37	37	19	50	−37	0.203	33	0	51	41	27	56	−25	0.157
Soups, sauces and miscellaneous	53	0	113	60	14	101	−11	0.044	65	0	111	75	14	120	−15	0.225
Nuts, seeds, herbs and spices	5	0	11	6	0	12	−11	0.500	7	101	14	6	0	12	10	0.744
Chocolate confectionery	11	0	24	12	0	24	−3	0.938	13	208	23	15	0	26	−14	0.739
Non– chocolate confectionery	4	0	10	8	0	29	−136	0.354	9	0	24	13	0	27	−40	0.201
Savoury snacks	7	0	20	9	295	23	−27	0.745	4	159	12	7	0	15	−50	0.434
Alcoholic beverages	163	0	592	196	0	709	−20	0.592	64	0	206	85	0	260	−32	0.653
Teas	353	250	406	533	295	691	−51	0.129	340	250	427	506	250	767	−49	0.282
Coffees	176	0	269	201	0	303	−14	0.592	173	0	228	244	160	356	−41	0.280
Carbonated beverages	96	0	298	92	0	247	4	0.701	129	0	383	106	0	320	17	0.895
Squashes and cordials	25	0	130	44	0	172	−75	0.317	58	0	323	47	0	225	19	0.495
Water	753	316	990	1405	1136	1265	−87	0.000	787	568	832	1309	1136	986	−66	0.000

^ⱡ^ Calculated as the difference of the mean intake (self-administered- interviewer-led) divided by the mean intake (self-administered) and multiplied by 100.

**Table 5:** Association between estimates of food group intake from self-administered and interviewer-led recalls split by recall number.

Food Groups	Recall 1							Recall 2						
	Spearman Correlations		% Classified in the Same Tertile	% Classified in Opposite Tertile	p *	Mean Difference	Limits of Agreement ^†^	Spearman Correlations		% Classified in the Same Tertile	% Classified in Opposite Tertile	p *	Mean Difference	Limits of Agreement ^†^
	*r*	*p*	%	%				*r*	*p*	%	%			
Grains, rice, pastas and savouries	0.677	<0.001	77.2	10.1	0.010	33.01	7.96/58.06	0.743	<0.001	75.9	10.1	0.298	8.43	−7.60/24.46
Bread and rolls	0.837	<0.001	74.7	3.8	0.003	−15.32	−25.34/–5.29	0.780	<0.001	68.4	1.3	<0.001	−21.73	−33.14/−10.33
Breakfast cereals	0.926	<0.001	93.7	0.0	0.213	28.01	−16.36/72.38	0.950	<0.001	92.4	1.3	0.448	13.43	−21.66/48.52
Biscuits, cakes and pastries	0.835	<0.001	84.8	3.8	0.039	−7.68	−14.97/–0.40	0.873	<0.001	84.8	1.3	0.230	−4.22	−11.15/2.72
Milk and yoghurt	0.813	<0.001	78.5	5.0	0.032	−46.28	−88.58/–3.98	0.665	<0.001	75.9	11.4	0.079	−35.67	−75.60/4.26
Milks	0.788	<0.001	81.0	7.6	0.050	−41.11	−82.23/0.00	0.684	<0.001	74.7	11.4	0.119	−32.51	−73.60/8.59
Creams, ice creams and chilled desserts	0.850	<0.001	93.7	0.0	0.226	−7.31	−19.24/4.62	0.595	<0.001	91.1	0.0	0.587	2.28	−6.03/10.59
Cheeses	0.714	<0.001	24.1	2.5	0.041	−4.42	−8.65/–0.19	0.729	<0.001	25.3	0.0	0.001	−8.68	−13.55/−3.82
Butter, spreading fats and oils	0.782	<0.001	75.9	5.1	0.083	−2.28	−4.88/0.31	0.762	<0.001	74.7	5.1	0.007	−5.13	−8.85/−1.42
Other dairy	0.880	<0.001	79.7	3.8	0.012	−13.71	−24.36/–3.06	0.795	<0.001	74.7	6.4	0.014	−11.35	−20.34/−2.37
Eggs and egg dishes	0.904	<0.001	29.1	0.0	0.020	−6.99	−12.85/–1.12	0.909	<0.001	96.2	0.0	0.733	1.00	−4.80/6.80
Potatoes and potato products	0.886	<0.001	88.6	3.8	0.851	−1.13	−13.02/10.76	0.921	<0.001	96.2	2.6	0.478	−3.47	−13.15/6.22
Veg and veg dishes	0.838	<0.001	88.6	0.0	0.366	−8.39	−26.79/10.0	0.841	<0.001	78.5	2.6	0.035	−16.59	−31.96/−1.23
Fruit and fruit juices	0.760	<0.001	73.4	3.8	0.593	−13.38	−62.95/36.19	0.848	<0.001	73.4	2.5	0.193	19.81	−10.24/49.86
Fish and fish dishes	0.052	<0.001	100.0	0.0	0.609	1.25	−3.61/6.12	0.906	<0.001	96.2	0.0	0.510	−1.58	−6.34/3.18
Meat and meat products	0.768	<0.001	67.1	5.1	0.101	−20.34	−44.75/4.07	0.824	<0.001	73.4	2.6	0.443	8.41	−13.29/30.10
Sugars, confectionery, preserves and savoury snacks	0.848	<0.001	79.7	2.6	0.009	−10.20	−17.72/–2.67	0.814	<0.001	69.6	3.8	0.011	−8.33	−14.72/−1.94
Soups, sauces and miscellaneous	0.604	<0.001	67.1	13.9	0.502	−6.08	−24.05/11.88	0.675	<0.001	70.9	10.1	0.272	−9.74	−27.28/7.80
Nuts, seeds, herbs and spices	0.734	<0.001	25.3	3.8	0.545	−0.59	−2.54/1.35	0.831	<0.001	26.6	2.5	0.332	0.68	−0.71/2.08
Chocolate confectionery	0.876	<0.001	94.9	0.0	0.724	−0.38	−2.52/1.76	0.858	<0.001	30.4	1.3	0.393	−1.83	−6.06/2.41
Non-chocolate confectionery	0.815	<0.001	94.9	0.0	0.119	−4.82	−10.91/1.26	0.705	<0.001	88.6	0.0	0.044	−3.61	−7.12/−0.10
Savoury snacks	0.940	<0.001	98.7	0.0	0.313	−1.82	−5.39/1.75	0.865	<0.001	96.2	0.0	0.010	−2.23	−3.91/−0.54
Alcoholic beverages	0.906	<0.001	96.2	0.0	0.216	−32.25	−83.76/19.26	0.918	<0.001	97.5	0.0	0.032	−20.81	−39.80/−1.82
Teas	0.837	<0.001	74.7	1.3	0.004	−179.56	−301.51/–57.60	0.838	<0.001	88.6	3.8	0.012	−166.39	−295.94/−36.85
Coffees	0.941	<0.001	86.1	1.3	0.018	−24.43	−44.48/–4.38	0.796	<0.001	73.4	6.3	0.008	−70.95	−122.98/−18.92
Carbonated beverages	0.793	<0.001	94.9	0.0	0.899	3.70	−54.06/61.45	0.785	<0.001	93.7	2.5	0.360	22.41	−25.99/70.80
Squashes and cordials	0.714	<0.001	96.2	0.0	0.124	−18.78	−42.81/5.25	0.784	<0.001	97.5	0.0	0.567	10.78	−26.56/48.13
Water	0.490	<0.001	55.7	11.4	<0.001	−652.41	−924.93/–379.88	0.461	<0.001	43.0	0.0	<0.001	−522.18	−732.58/−311.77

^†^ Lower and upper limits of agreement (mean difference ± 2 SD).

**Table 6:** Incidence of matches, omission, and intrusions observed between the self-administered and interviewer administered methods in the reporting of individual food and drink items.

	Total Match ^a^	Exact Matches	Omissions	Intrusions
Recall 1	84%	69%	12%	4%
Recall 2	86%	68%	11%	3%
Females ^b^	86%	71%	10%	4%
Males ^b^	84%	67%	13%	3%

^a^ Total match includes exact matches and approximate matches. An example of an approximate match is skimmed milk listed using one method and semi-skimmed milk in the other method; ^b^ Calculated using the average of both recalls.
